# The systematic analysis and 10-year prediction on disease burden of childhood cancer in China

**DOI:** 10.3389/fpubh.2022.908955

**Published:** 2022-09-06

**Authors:** Bo Zhu, Xiaomei Wu, Wenxiu An, Bing Yao, Yefu Liu

**Affiliations:** ^1^Department of Neurosurgery, Cancer Hospital of China Medical University/Liaoning Cancer Hospital and Institute, Shenyang, China; ^2^Department of Clinical Epidemiology and Center of Evidence Based Medicine, The First Hospital of China Medical University, Shenyang, China; ^3^Department of Medical Management, Cancer Hospital of China Medical University/Liaoning Cancer Hospital and Institute, Shenyang, China; ^4^Department of Hepatobiliary Surgery, Cancer Hospital of China Medical University/Liaoning Cancer Hospital and Institute, Shenyang, China

**Keywords:** childhood cancer, epidemiology, cancer burden, systematic analysis, GBD

## Abstract

**Background:**

There is a lack of in-depth analysis regarding the disease burden of childhood cancer in China. Indeed, this is the first time the topic has been addressed in detail. Drawing on population-based data for the past 30 years, this study systematically analyzes the composition and long-term trend of this disease burden in China.

**Methods:**

GBD 2019 contained population-based data from 1990 to 2019 and was prepared using Microsoft Excel 2016. We used AAPC and ARIMA models for trend analysis and prediction formulation.

**Results:**

In 2019, there were 45,601 new cases, 9,156 cancer deaths, and 782,530 DALYs in China. From 1990 to 2019, leukemia, together with brain and CNS cancer, invariably ranked highest in terms of new cases, cancer deaths, and DALYs. Leukemia accounted for more than 50%, but decreased over time. By contrast, the proportions for brain and CNS cancer increased. There were significant decreases in the overall incidence, mortality, and DALY rates in China, but these were still higher than the corresponding global average levels. Considering all types of childhood cancer, the incidence rate of testicular cancer showed the biggest increase, and the mortality and DALY rates of leukemia showed the largest decrease. In terms of different age groups, the overall incidence rate of childhood cancers increased in 0 to 4 age group, but it decreased in 5 to 14 age groups. The overall mortality and DALY rates of childhood cancers decreased in all four age groups. Over the next 10 years, the overall incidence rate of childhood cancer will increase, but the overall mortality and DALY rates will decrease. The increase in malignant skin melanoma will comprise the largest rise in the incidence, while the decrease for leukemia will be the largest fall in the incidence, cancer deaths, and DALYs.

**Conclusion:**

The disease burden of all childhood cancers in China remains highly serious, especially for certain types of cancer and certain age groups. China should focus more emphatically on the incidence of childhood cancer in future, and it must consistently strengthen investment in the relevant research and medical resources to reduce the disease burden in this field.

## Introduction

The more common types of childhood cancer are different from the commonest adult cancers, and the histological types are unique (1). In addition to the physiological, psychological, and social pressures brought by cancer, children with malignant tumors may also face growth and developmental disorders, gonadal dysfunction, and the long-term adverse effects of treatment, such as organ function damage and secondary tumors (2). Childhood cancer recently ranked sixth in total cancer burden and ninth in childhood disease burden in the world (3). The US Centers for Disease Control and Prevention reported that the average incidence rate of childhood and adolescent cancer was 186.6 per million in 2014. About one in 285 children will be diagnosed with cancer before age 20, and about one in 530 young people aged 20 to 39 are childhood cancer survivors (4).

Developed countries have made significant progress in diagnosing and treating childhood cancer. The 5-year survival rate of childhood cancer is about 80% (1). Only 10% of childhood cancers occur in developed countries, and consequently, nearly 90% of childhood cancer patients are in developing/underdeveloped countries (5). These individuals, however, often cannot receive timely diagnosis and high-level treatment, and they evince a disproportionately high number of person-years of life lost. Childhood cancer has caused, and causes, great harm to patients themselves and their families (5). At the same time, the whole treatment cycle of childhood cancer is so long, and medical costs are so high that a substantial economic burden to the country is generated that needs continuous social attention (6).

Cancer prevention and treatment initiatives should address all age groups. Understanding the epidemiological characteristics of childhood cancer can provide valuable clues for relevant academic research. It also provides a scientific basis for the formulation of childhood cancer prevention and control strategies, as well as the construction of prevention and control systems. Worldwide, Asia and Oceania have evinced the highest disease burden for childhood cancer. Specifically, India ranks first, and China ranks second in the world (3). China is undergoing a rapid epidemiological transformation, shifting from infectious diseases to non-communicable diseases (7). Most previous studies on childhood cancer in China have been based on a single cancer registry in a particular city or county, and there are few reports comprising systematic analysis of the epidemiology of all childhood cancers and/or predictions for these conditions (8, 9). Therefore, our study analyzed the GBD data to explore the distribution characteristics of childhood cancer from 1990 to 2019, while forecasting the relevant 10-year trend in China.

## Methods

### Design and data source

GBD 2019 analyzed the disease burden of 369 diseases and injuries, globally, in 204 countries and regions. It also comprehensively estimated and evaluated 369 diseases or injuries, in 34 provincial administrative units in China, using standard unified criteria. Via comparable methods (10, 11), GBD 2019 used ICD-9 and ICD-10 to classify diseases, and it reasonably redistributed garbage codes to make the estimation results more accurate. The specific methods are reported in the literature (11).

### Data collection

GBD 2019 comprised 31 cancer types. The World Health Organization (WHO) defines childhood cancer as occurring between birth and 14 years of age. By querying the GBD database, we found that the latter contained 14 types of childhood cancer, as follows: liver cancer; tracheal, bronchus, and lung cancer (“lung cancer” in brief); colon and rectum cancer; lip and oral cavity cancer; nasopharynx cancer; malignant skin melanoma; ovarian cancer; testicular cancer; kidney cancer; brain and central nervous system (CNS) cancer; thyroid cancer; Hodgkin lymphoma; non-Hodgkin lymphoma; and leukemia (3). As with previously published articles, the related data are available to the public and can be extracted via the GBD Results Tool (http://ghdx.healthdata.org/gbd-results-tool) (12, 13).

We collected the indicators of cancer incidence, death, and disease burden among China's 0 to 14 age-group population. Case fatality is a measure of the severity of disease. The mortality-to-incidence ratio value (MI value) was calculated via the incidence and death data provided by the GBD database (14). We collected the overall relevant indicators for the 0 to14 age group and included the relevant indicators of different age groups (i.e., the <1 year, 1–4, 5–9, and 10–14 age groups). We also extracted global data for comparison. All extracted data were utilized according to the GBD Protocol.

### Statistical analysis

All data were prepared using Microsoft Excel 2016, and all the statistical analyses were performed using R software (R version 4.1.1). The annual percentage change (APC) was used to evaluate the trend of childhood cancer incidence, death, and disease burden for the past 30 years. The calculation process for APC was divided into two steps: (1) y= α + β x+ ε, where y = ln (rate), x = year, and ε was the error term; (2) APC=100^*^[exp(β)- 1].

When APC and its 95% confidence interval (CI) were higher than zero, this showed an upward trend; conversely, when APC and its 95% CI were lower than zero, this reflected a downward trend. For predictive purposes, the autoregressive integrated moving average (ARIMA) model was generally used for non-stationary data, in the context of the long-term trend, to forecast the incidence, death, and disability adjusted of life year (DALY) rates for the next 10 years. Three steps, namely (1) a stationarity test, (2) model identification and order determination, and (3) model diagnosis, were used to fit the best ARIMA model. We obtained all possible values of model parameters through the autocorrelation function (ACF) and partial autocorrelation function (PCAF). Finally, all the obtained data were fitted and verified by simultaneous automatic fitting, and the best model was selected by the Akaike information criterion (AIC) and determination coefficient R^2^.

## Results

### The number of new cases, cancer deaths, and DALYs for Childhood cancers in China from 1990 to 2019

In 2019, there were 45,601 new cases, 9,156 cancer deaths, and 782,530 DALYs due to childhood cancers in China. The leading five conditions in terms of new cases were leukemia (27,727), brain and CNS cancer (7,966), testicular cancer (3,010), non-Hodgkin lymphoma (2,372), and kidney cancer (2,223). The top five regarding cancer deaths, meanwhile, were leukemia (4806), brain and CNS cancer (2816), non-Hodgkin lymphoma (584), liver cancer (368), and kidney cancer (313). The leading five in terms of DALYs were the same as those for cancer deaths.

From 1990 to 2019, the number of all new cases fell from 104,543 to 45,602, and this decrease of 56.38% in China was higher than the global average of 29.25%. In the PRC, indeed, only three types of childhood cancer increased in terms of occurrence, namely testicular cancer (152.31%), malignant skin melanoma (120.20%), and thyroid cancer (35.57%). Compared to the situation globally, nevertheless, the percentage increases in testicular cancer and malignant skin melanoma were higher in China. Conversely, the number of all childhood cancer deaths fell from 37,477 to 9,156, comprising a decrease of 70.18%. All types of childhood cancer in China showed a downward trend, and the percentage decrease of Hodgkin lymphoma was the highest (-90.71%). Compared to the worldwide situation, the percentage decreases in all types of childhood cancer were higher in China; the changes in the number of DALYs were similar to those for cancer deaths (Supplementary Table S1).

### The proportions of new cases, cancer deaths, and DALYs for childhood cancers in China from 1990 to 2019

**Figure 1:**
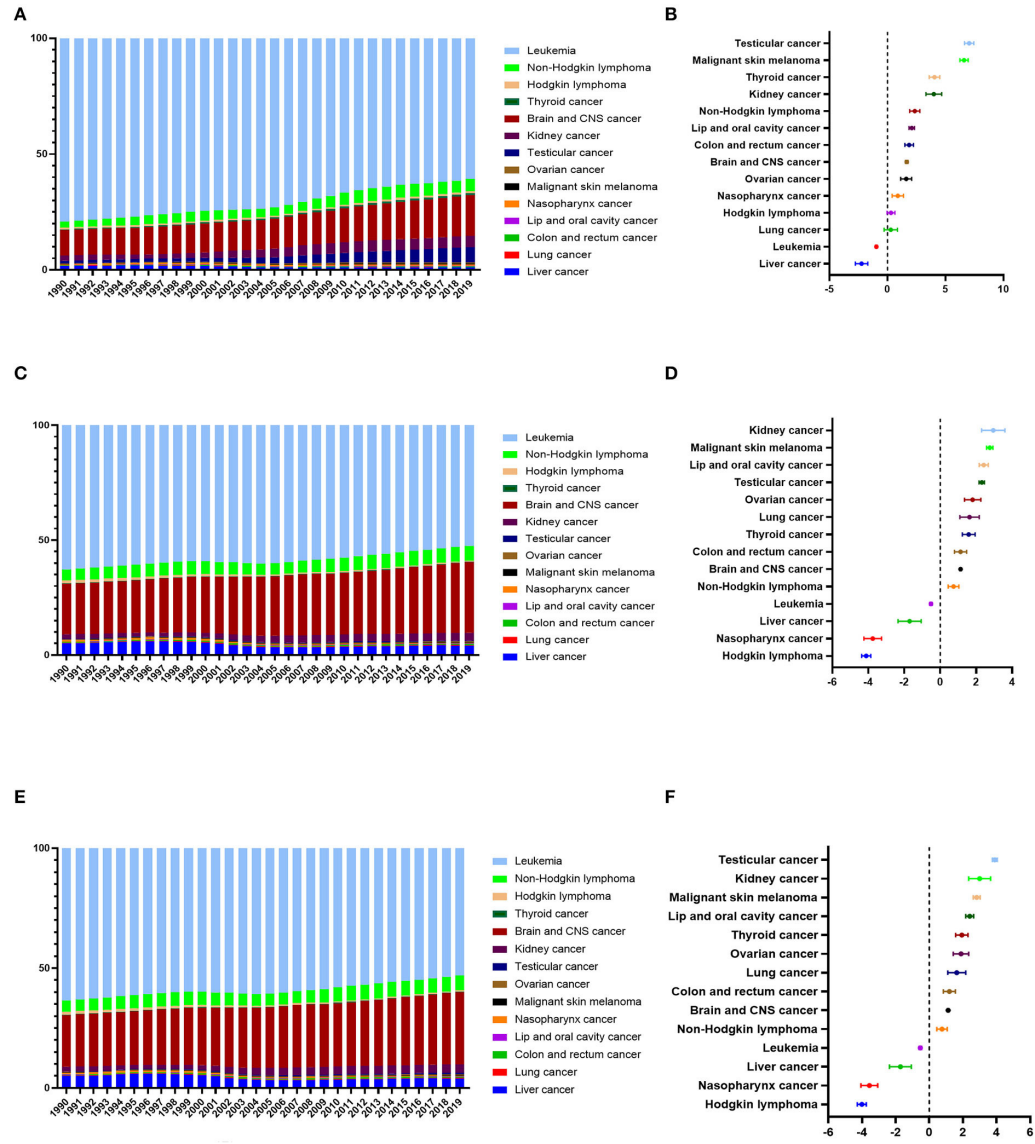
Changes in the proportion of new cases, cancer deaths, and DALYs of all the childhood cancers in China from 1990 to 2019. **(A)** The proportion of new cases of all the childhood cancers in China from 1990 to 2019. **(B)** The average annual percentage change (AAPC) in the proportion of new cases of all the childhood cancers in China from 1990 to 2019. **(C)** The proportion of cancer deaths of all the childhood cancers in China from 1990 to 2019. **(D)** The average annual percentage change (AAPC) in the proportion of cancer deaths of all the childhood cancers in China from 1990 to 2019. **(E)** The proportion of DALYs of all the childhood cancers in China from 1990 to 2019. **(F)** The average annual percentage change (AAPC) in the proportion of DALYs of all the childhood cancers in China from 1990 to 2019.

We further analyzed the proportional trends of new cases, cancer deaths, and DALYs for all the main childhood cancers in China. The top five proportional increases of new-case childhood cancer were for testicular cancer, malignant skin melanoma, thyroid cancer, kidney cancer, and non-Hodgkin lymphoma. Only the proportions for leukemia and liver cancer evinced a downward trend (Figures 1A,B). The top five increases in the proportion of childhood cancers linked to cancer deaths were kidney cancer, malignant skin melanoma, lip / oral cavity cancer, testicular cancer, and ovarian cancer. Meanwhile, the relevant proportions for four types of childhood cancer (leukemia, liver cancer, nasopharynx cancer, and Hodgkin lymphoma) showed a downward trend (Figures 1C,D). Regarding DALYs, the top five increases in childhood cancer proportions were for testicular cancer, kidney cancer, malignant skin melanoma, lip and oral cavity cancer, and thyroid cancer. Meanwhile, the proportions for four types of childhood cancer (namely, leukemia, liver cancer, nasopharynx cancer, and Hodgkin lymphoma) showed a downward trend (Figures 1E,F).

In terms of new cases, cancer deaths, and DALYs, the proportions for leukemia always ranked first, accounting, respectively, for 60.80, 52.49, and 53.04% in 2019. Still, leukemia proportions did show a downward trend. Meanwhile, the proportions regarding brain / CNS cancer always ranked second, and these showed an upward trend. They accounted for 17.47% (new cases), 30.76% (cancer deaths), and 30.17% (DALYs) in 2019.

### The incidence, mortality, and DALY rates of childhood cancers in China from 1990 to 2019

In 2019, China's overall incidence, mortality, and DALY rates for childhood cancers were 20.29, 4.07, and 348.13 per 100,000, respectively. Across all 14 childhood cancers discussed here, leukemia and brain / CNS cancer were ranked as the first two in incidence, mortality, and DALY rates.

**Figure 2:**
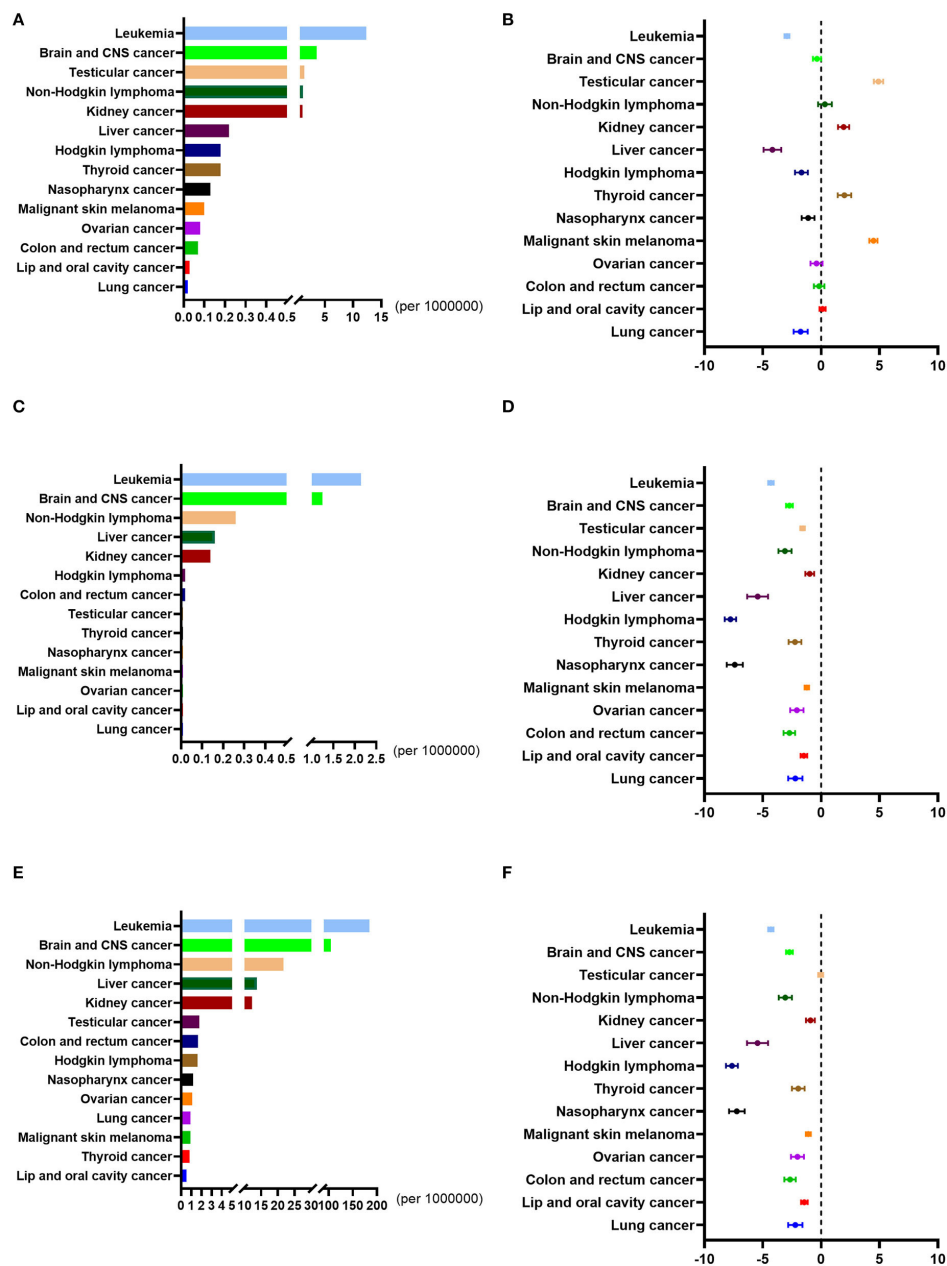
Incidence rates, mortality rates, and DALY rates of all the childhood cancers in China from 1990 to 2019. **(A)** The incidence rates of all the childhood cancers in China in 2019. **(B)** The average annual percentage change (AAPC) in the incidence rates of all the childhood cancers in China from 1990 to 2019. **(C)** The mortality rates of all the childhood cancers in China in 2019. **(D)** The average annual percentage change (AAPC) in the mortality rates of all the childhood cancers in China from 1990 to 2019. **(E)** The DALY rates of all the childhood cancers in China in 2019. **(F)** The average annual percentage change (AAPC) in the DALY rates of all the childhood cancers in China from 1990 to 2019.

From 1990 to 2019, the overall incidence rate of childhood cancer decreased from 32.38 to 20.29 per 100,000, with average APC (AAPC) being −0.34 (95%CI: −0.41 to −0.27), which was lower than the global AAPC (−0.47, 95%CI: −0.54 to −0.40). Still, the overall incidence rate of childhood cancer in China was higher than the global average. The incidence rates of four childhood cancers in China showed an upward trend, and these were testicular cancer (AAPC: 4.92, 95%CI: 4.51 to 5.32), malignant skin melanoma (AAPC: 4.48, 95%CI: 4.13 to 4.83), thyroid cancer (AAPC: 1.99, 95%CI: 1.42 to 2.57) and kidney cancer (AAPC: 1.92, 95%CI: 1.45 to 2.40). All these were higher than the corresponding global cancer rates. The overall mortality rate of childhood cancer, nonetheless, decreased from 10.99 per 100,000 to 4.07 per 100,000, with AAPC being −3.75 (95%CI: −4.05 to −3.46), this being higher than the global AAPC (−1.91, 95%CI: −2.02 to −1.81). The overall mortality rate of childhood cancer in China was higher than the global average. This was true, even though the mortality rate of all childhood cancers in China evinced a downward trend, and leukemia showed the biggest decrease. The AAPC for each type of childhood cancer was lower than the corresponding global cancer figure. The changes in the DALY rates, meanwhile, were similar to those of the mortality rates (Figure 2; Supplementary Table S2).

### The case fatality of childhood cancers in China from 1990 to 2019

In 2019, the MI values of six childhood cancers were higher, and the MI values of eight childhood cancers were lower, than the average MI value of 0.2. From 1990 to 2019, the MI values of all childhood cancers in China showed a downward trend. The percentage decreases in the MI value of each childhood cancer in China were higher than the global average percentage decreases for that cancer (Supplementary Table S3).

### Variations in childhood cancers in different age groups (<1 year, 1 to 4, 5 to 9, and 10 to 14) in China from 1990 to 2019

In 2019, as shown in Supplementary Table S4, there were 6,763 new cases, 1,311 cancer deaths, and 119,731 DALYs in the < 1 year age group, which contained six types of cancer. Meanwhile, there were 16,923 new cases, 2,715 cancer deaths, and 203,013 DALYs in the 1 to 4 age group, which contained seven types of cancers. There were 13,388 new cases, 2,861 cancer deaths, and 241,552 DALYs in the 5 to 9 age group, which comprised 13 types of cancer aside from lung cancer. Finally, there were 8,528 new cases, 2,268 cancer deaths, and 178,234 DALYs in the 10–14 age group, which contained 14 types of cancers.

**Figure 3:**
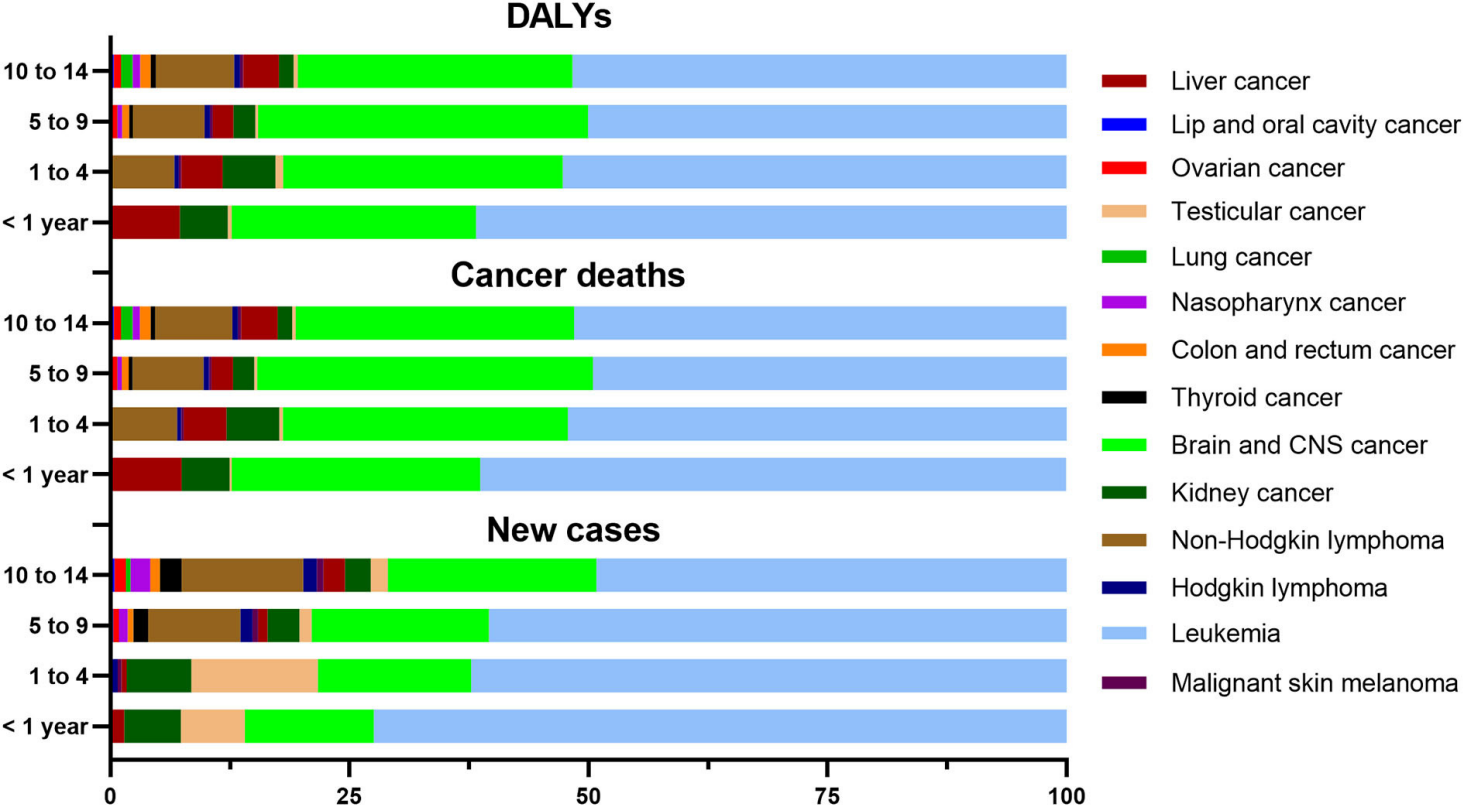
Proportion of new cases, cancer deaths, and DALYs of childhood cancers in four age groups in China in 2019.

For new cases, cancer deaths, and DALYs, the proportions for leukemia and brain / CNS cancer always ranked as the leading two for each age group (Supplementary Figures S1, S2, S3). The proportion for leukemia decreased with age, while that for brain and CNS cancer increased with age (Figure 3). The distribution of new cases of testicular cancer was mainly concentrated in the 1 to 4 age group, and the distribution of cancer deaths and DALYs was similar for that condition. The distribution of new cases, cancer deaths, and DALYs for the other childhood cancers was similar for the four age groups (Supplementary Figure S4).

**Figure 4:**
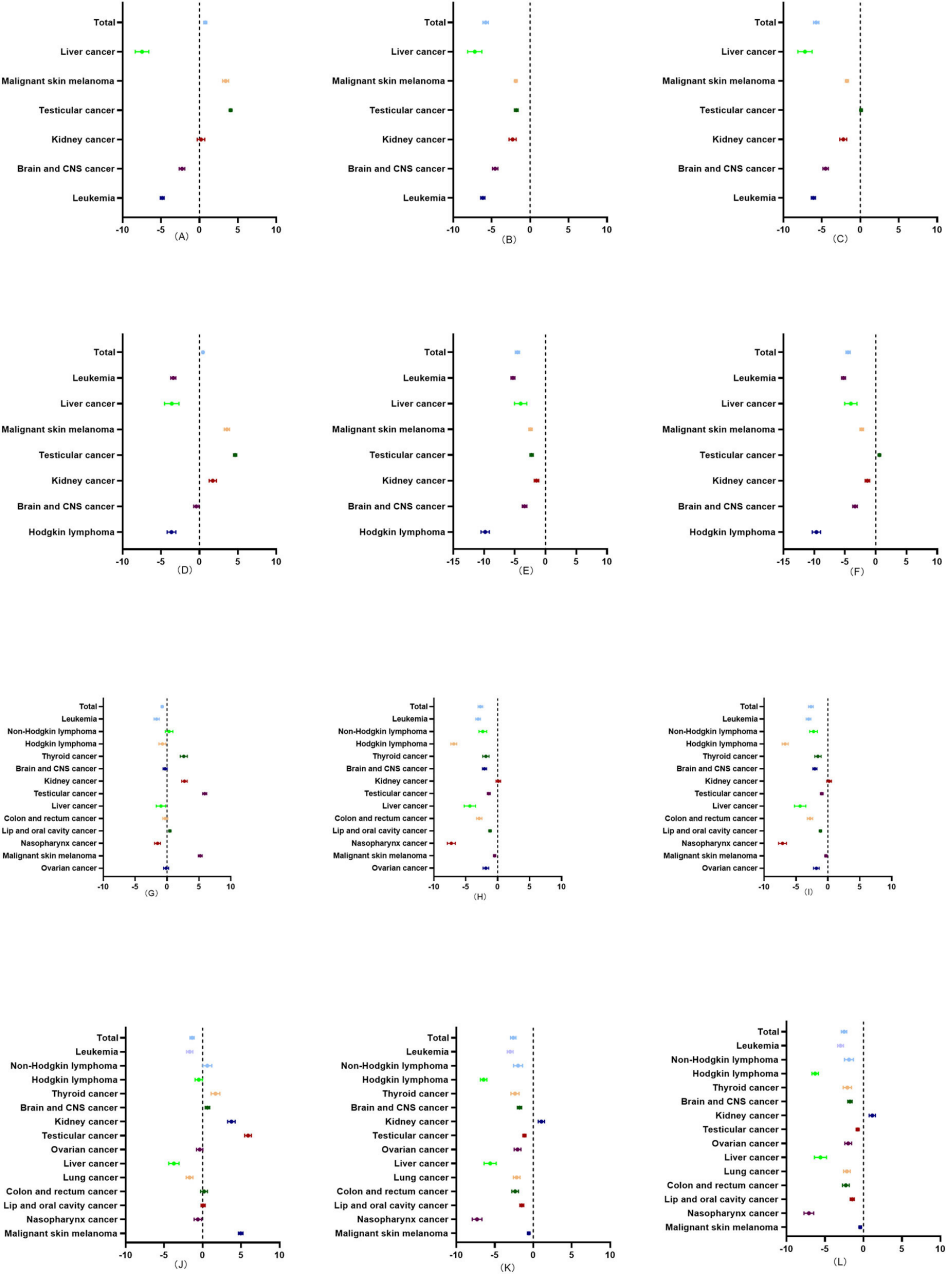
Average annual percentage change (AAPC) in the incidence, mortality, and DALY rates of childhood cancers in four age groups (<1 year, 1–4, 5–9, and 10–14) in China from 1990 to 2019. **(A)** The AAPC in the incidence rate of childhood cancers in <1 year age group in China from 1990 to 2019. **(B)** The AAPC in the mortality rate of childhood cancers in <1 year age group in China from 1990 to 2019. **(C)** The AAPC in the DALY rate of childhood cancers in <1 year age group in China from 1990 to 2019. **(D)** The AAPC in the incidence rate of childhood cancers in 1–4 age group in China from 1990 to 2019. **(E)** The AAPC in the mortality rate of childhood cancers in 1–4 age group in China from 1990 to 2019. **(F)** The AAPC in the DALY rate of childhood cancers in 1 to 4 age group in China from 1990 to 2019. **(G)** The AAPC in the incidence rate of childhood cancers in the 5 to 9 age group in China from 1990 to 2019. **(H)** The AAPC in the mortality rate of childhood cancers in 5–9 age group in China from 1990 to 2019. **(I)** The AAPC in the DALY rate of childhood cancers in the 5–9 age group in China from 1990 to 2019. **(J)** The AAPC in the incidence rate of childhood cancers in the 10 to 14 age group in China from 1990 to 2019. **(K)** The AAPC in the mortality rate of childhood cancers in the 10–14 age group in China from 1990 to 2019. **(L)** The AAPC in the DALY rate of childhood cancers in the 10 to 14 age group in China from 1990 to 2019.

From 1990 to 2019, the overall incidence rate of childhood cancers evinced an upward trend in the <1 year and 1 to 4 age groups, but a downward trend in the 5 to 9 and 10 to 14 age groups. The incidence rates of malignant skin melanoma and testicular cancer invariably showed an upward trend, while the incidence rate of leukemia showed a continual downward trend for the four age groups. The overall mortality rates of childhood cancers also showed a downward trend for the four age groups. The mortality rate of kidney cancer, meanwhile, showed a downward trend in the <1 year and 1 to 4 age groups, no obvious changes in the 5–9 age group, and an upward trend in the 10–14 age group. The changes in the DALY rates were similar to those of the mortality rates in the four age groups (Figure 4).

As shown in Supplementary Table S5, the MI values for leukemia were lower than the average decrease in the four age groups from 1990 to 2019.

### The prediction trend regarding childhood cancers in China for the next 10 years

Over the next 10 years, the overall incidence rate of childhood cancer will slightly increase (AAPC: 0.01, 95%CI: 0.00 to 0.03), but the overall mortality and DALY rates of childhood cancer will decrease (AAPC in mortality: −6.87, 95%CI: −7.45 to −6.29; AAPC in DALYs: −6.76, 95%CI: −7.33 to −6.20).

**Figure 5:**
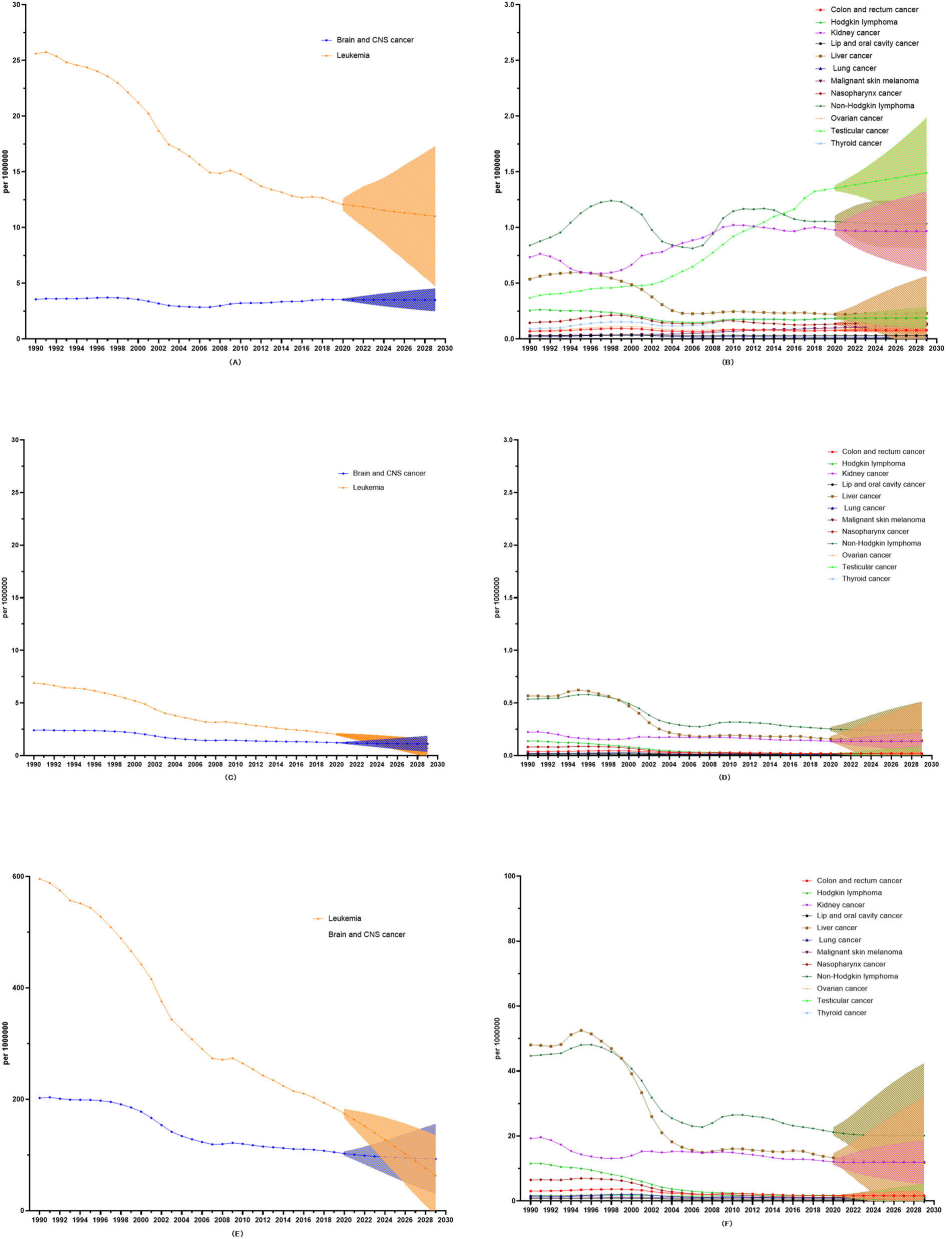
10-year prediction of incidence, mortality, and DALY rates in all the childhood cancers. **(A)** The 10-year prediction of incidence rates in leukemia and Brain and CNS cancer. **(B)** The 10-year prediction of incidence rates in all the other childhood cancers. **(C)** The 10-year prediction of mortality rates in leukemia and Brain and CNS cancer. **(D)** The 10-year prediction of mortality rates in all the other childhood cancers. **(E)** The 10-year prediction of DALY rates in leukemia and Brain and CNS cancer. **(F)** The 10-year prediction of DALY rates in all the other childhood cancers.

As regards incidence, mortality, and DALY rates, four childhood cancers (leukemia, brain and CNS cancer, non-Hodgkin lymphoma, and lung cancer) show a downward trend, and only nasopharynx cancer evinces an upward trend in incidence, mortality, and DALY rates. Among all 14 types of childhood cancer, leukemia shows the most significant predictive decrease *via* three indicators [AAPC in incidence: −1.03 (−1.10 to −0.97), AAPC in mortality: −10.66 (−12.02 to −9.27), and AAPC in DALYs: −10.47 (−11.79 to −9.12)). Conversely, malignant skin melanoma shows the most significant predictive increase in incidence rate (AAPC: 2.43 (2.37 to 2.49)] (Figure 5; Table 1).

**Table 1:** Average annual percentage change (AAPC) in the 10-year prediction of incidence, mortality, and DALY rates in all the childhood cancers.

**Cancer type**	**Incidence**	**Mortality**	**DALYs**
Brain and CNS cancer	−0.05 (−0.07 to −0.03)	−1.04 (−1.23 to −0.86)	−1.08 (−1.26 to −0.89)
Colon and rectum cancer	0.35 (0.06 to 0.64)	−0.23 (−0.31 to −0.14)	−0.21 (−0.29 to −0.13)
Hodgkin lymphoma	0.13 (0.06 to 0.19)	−2.89 (−2.98 to −2.81)	−2.54 (−2.61 to −2.47)
Kidney cancer	−0.10 (−0.16 to −0.03)	−0.19 (−0.30 to −0.07)	0.00 (−0.12 to 0.12)
Leukemia	−1.03 (−1.10 to −0.97)	−10.66 (−12.02 to −9.27)	−10.47 (−11.79 to −9.12)
Lip and oral cavity cancer	−0.11 (−0.13 to −0.09)	0.04 (0.02 to 0.06)	0.03 (0.01 to 0.05)
Liver cancer	0.43 (0.27 to 0.59)	−1.15 (−1.41 to −0.89)	−1.20 (−1.48 to −0.93)
Lung cancer	−0.04 (−0.06 to −0.01)	−0.07 (−0.12 to −0.03)	−0.07 (−0.12 to −0.03)
Malignant skin melanoma	2.43 (2.37 to 2.49)	−1.38 (−1.40 to −1.36)	−1.20 (−1.21 to −1.19)
Nasopharynx cancer	0.42 (0.21 to 0.63)	0.09 (0.07 to 0.11)	0.14 (0.10 to 0.18)
Non–Hodgkin lymphoma	−0.22 (−0.34 to −0.11)	−0.46 (−0.72 to −0.19)	−0.50 (−0.77 to −0.22)
Ovarian cancer	0.41 (−0.06 to 0.88)	0.04 (0.02 to 0.05)	0.05 (0.03 to 0.06)
Testicular cancer	1.08 (1.07 to 1.09)	−0.16 (−0.22 to −0.09)	−0.55 (−0.73 to −0.36)
Thyroid cancer	0.12 (0.04 to 0.20)	−0.28 (−0.36 to −0.19)	−0.19 (−0.25 to −0.13)
Total	0.01 (0.00 to 0.03)	−6.87 (−7.45 to −6.29)	−6.76 (−7.33 to −6.20)

## Discussion

On an international scale, the incidence rate of childhood cancers varied greatly among different countries, ranging from 130 cases per million children (British Isles) to 160 cases (the Scandinavian countries). During the last 30 years, the incidence rate of childhood cancers has increased, but it has also varied among different subtypes of childhood cancer. Therefore, it remains critical to understand the precise situation regarding childhood cancers in China. The most comprehensive analysis (thus far) of the disease burden of childhood cancer in that country, from 1990 to 2019, has now been conducted. Most previous studies focused on incidence and mortality rates, which could not comprehensively reflect the burden of childhood cancer (15, 16). In our study, therefore, we further introduced DALYs and case fatalities as indicators, since this can provide a useful summary of early and treatment-related mortality, while offering a more comprehensive and lifelong assessment of the burden of childhood cancer (17).

Overall, in 2019, DALY rates were detected at 17.15 and 85.53 times the incidence and mortality rates, respectively. This result, which was consistent with the global childhood disease burden, showed that, although rates of childhood cancer incidence and mortality were relatively low in China, the disease burden of childhood cancer was huge. From 1990 to 2019, nonetheless, the overall numbers and rates of incidence, mortality, and DALYs for childhood cancer in China decreased significantly, with the decrease in case fatality also being higher than the global average. These results indicate that China has made significant progress in preventing and treating childhood cancer in the past 30 years.

Leukemia, which still accounted for more than 50% of new cases, cancer deaths, and DALYs by 2019, always ranked first. This remained the most important malignant condition among Chinese children, although its incidence, mortality, and DALY rates showed a downward trend from 1990 to 2019. The new cases, cancer deaths, and DALYs of leukemia were mainly concentrated in the 1 to 9 age groups. At present, childhood leukemia is principally treated via combined chemotherapy. According to the large-sample study for the ALL clinical research program launched by the China Children's Cancer Professional Committee in 2015 (18), the 5-year overall survival rate of ALL may reach 91.1%, which would already meet the goal of the WHO Global Initiative on childhood cancer (19). Despite the exact etiology of childhood leukemia remaining unclear (20), it seems that under China's current medical, technical, and economic conditions, a better curative effect may be obtained if medical treatment is properly handled.

Our study also found that leukemia case fatality in China was lower than the global average in 2019, and it showed a downward trend from 1990 to 2019. The average survival rate for acute lymphoblastic leukemia (ALL) also increased by 10% in the 20 years from 1995 to 2014; nonetheless, survival was still below 60% in China (21). There was a large gap for domestic survival rates vs. those in developed countries (22, 23). There were also regional differences in the standardized treatment of childhood leukemia in China, and there was still much room for improvement in the standardized treatment of childhood leukemia in backward or poorly developed areas (24).

In China, brain and CNS cancer was the second most common type of cancer in children. Although its incidence, mortality, and DALY rates showed a downward trend, the proportion figures for brain and CNS cancer increased in the 30 years to 2019, in terms of new cases, cancer deaths, and DALYs; this was close to the levels of developed countries (25, 26). Reflecting the data from cancer registries, the results revealed important ethnic differences in the epidemiology of brain and CNS tumors in Hong Kong and the United States (27). Although 5-year age-standardized survival increased by 5–10% in China, the 5-year age-standardized survival rate was still only 41.1% in that country. Childhood ependymomas and choroid plexus tumors have an average survival rate of 76%, and medulloblastoma tumors have an average survival rate of 63%. Meanwhile, the survival rate varied widely for different subtypes of brain and CNS cancer (21). In future, we should pay attention to key subtypes, such as low-grade glioma, although the WHO has suggested that 5-year survival rates will reach 85% in 2030. Compared to other age groups, one should note a significant increase in the incidence rate in the 10–14 age group. This was perhaps because youngsters in the 10–14 age group could communicate with their parents, describe their situation, attract their parents' attention, and (thus) facilitate a timely diagnosis, so mortality and DALYs were relatively lower.

Lymphoma includes non-Hodgkin lymphoma and Hodgkin lymphoma. In 2019, the incidence of childhood lymphoma ranked fourth, and mortality and DALYs ranked third. In terms of incidence, mortality, and DALYs, non-Hodgkin lymphoma accounted for about 90%, while Hodgkin lymphoma accounted for less than 10%. Hodgkin lymphoma was more common in children over 5 years old, while non-Hodgkin lymphoma was more common in those over 10 years old. In the past 30 years, the incidence rates of non-Hodgkin lymphoma and Hodgkin lymphoma did not change significantly in the 5 to 14 age groups. In terms of mortality and DALYs, the decreases for non-Hodgkin lymphoma were lower than those for Hodgkin lymphoma. The conditions of most children with lymphoma progressed to stages III or IV, because lymphoma did not have specific clinical manifestations and could easily be misdiagnosed.

Indeed, the misdiagnosis rate of childhood lymphoma reached 58.6% (28). Therefore, the invasion scope of lymphoma expanded, which in turn affected the clinical-stage progression and prognosis. In developed countries, Hodgkin lymphoma has been recognized as a curable disease. Its survival rate has reached 90% in children, while the survival rate for non-Hodgkin lymphoma has also reached 80% in some jurisdictions, and this represents an increase compared with previous decades (29). By contrast, 5-year survival was only 61% in China—far behind developed countries (21). In the next 10 years, we find that although the mortality and DALYs of non-Hodgkin lymphoma will decrease significantly, the incidence will increase substantially. Therefore, one must focus on early diagnosis, and one must pay attention to improving the prognosis of children with lymphoma. Clinicians should evaluate the condition at different treatment stages, constantly improve the chemotherapy scheme, reduce the toxic and side effects caused by chemotherapy as much as possible, and (partly by these methods) achieve the best treatment effect for children.

We found that the incidence rate of testicular cancer and malignant skin melanoma increased significantly in the past 30 years. Indeed, it increased notably in all four age groups, especially in the 5 to 9 and 10 to 14 cohorts. By 2019, the incidence of childhood testicular cancer ranked third, as compared with the other conditions addressed here. Testicular cancer usually has a hidden onset. With the progress of medical technology and the popularization of related detection technologies, such as ultrasound, CT, and AFP, testicular cancer can be found and treated earlier, and both missed diagnosis and misdiagnosis rates can be reduced (30). Management of childhood testicular cancer differs from that for adults, due to different histological types and differing tolerances to chemotherapy drugs (31). Our results also found that the numbers and rates for testicular cancer mortality and DALYs in China fell significantly, which indicated that the level of testicular cancer treatment in China was acceptable.

Among all childhood cancers, the next 10 years will see the most significant increase in malignant skin melanoma. The 5-year survival rate is 58.8%, and the majority of malignant skin melanomas begin in the head, which clinicians should consider (32). Previously, the proportion of patients with recurrence was very high when malignant skin melanoma was diagnosed in its advanced stage, and most patients with recurrence died of related diseases. Therefore, the prevention and treatment of melanoma must also prioritize early detection, early diagnosis, and early treatment.

The GBD database provided comprehensive data. In fact, we conducted a detailed and in-depth analysis of Chinese data, but our study still had limitations. First, we used the GBD database to collect data on childhood cancer via the anatomical site-based cancer-type reporting system, which was highly effective for adult cancer. Nonetheless, morphology classification was crucial for the proper diagnosis and treatment of childhood cancer. In 2022, for the first time, childhood cancers were covered in a separate volume of the new (fifth) edition of the WHO classification of tumors (33). The current GBD classification system cannot fully express the disease burden of childhood cancer (3). More broadly, GBD offers a complete data collection and data collation system, and although there was a certain disjuncture with reality, we still considered the data reliable. Second, the assessment of DALYs in the GBD database was limited to 10 years after diagnosis. Children who survived over the past 10 years were considered to have the same incidence and mortality risk as the general population, but this hypothesis underestimated DALYs associated with childhood cancer (3). Third, the GBD data depended on the availability of reliable data from the various contributing countries. Consequently, although GBD had its own quality control methods, data insufficiency or low-quality data were further limitations. Conversely, compared with traditional cancer registration data, the GBD database contained more data sources via data collation, and this allowed for a comprehensive reflection of epidemic trend and disease burden.

## Conclusion

Treatment of childhood cancers represents a substantial cost, both for the families affected and for society. We must therefore strive fully to understand the incidence, death rates, and DALYs of childhood cancer and then take preventive measures to reduce the social and economic burden. From our results, it appears that overall incidence, death rates, and DALYs in childhood cancers have decreased significantly in the last 30 years, but this downward trend will itself decrease over the next decade. First, learning from the experience of the USA (4), we should set up an effective system for the prevention and treatment of childhood cancers, better to implement three-grade prevention. Second, some types of childhood cancer have received advanced forms of treatment, even by international standards; nonetheless, due to differences in regional, economic, and social factors (34), it is difficult to guarantee equality of healthcare, which is a key future goal. China must, therefore, prioritize and strengthen investment in research and medical resources pertaining to childhood cancers, to reduce the immense national disease burdens linked to these conditions.

## Data availability statement

The original contributions presented in the study are included in the article/Supplementary material, further inquiries can be directed to the corresponding authors.

## Author contributions

YL and BZ designed the whole research, XW and WA conducted the data collection, and BZ and XW analyzed the data. BY, BZ, and XW wrote the manuscript. All authors discussed the relevant results and approved the final manuscript.

## Funding

This study was partially supported by Shenyang Science and Technology Talent Program (RC210496).

## Conflict of interest

The authors declare that the research was conducted in the absence of any commercial or financial relationships that could be construed as a potential conflict of interest.

## Publisher's note

All claims expressed in this article are solely those of the authors and do not necessarily represent those of their affiliated organizations, or those of the publisher, the editors and the reviewers. Any product that may be evaluated in this article, or claim that may be made by its manufacturer, is not guaranteed or endorsed by the publisher.
