# Supplementary material for: The systematic analysis and 10-year prediction on disease burden of childhood cancer in China
Source: Front Public Health. 2022 Sep 6;10:908955. doi: 10.3389/fpubh.2022.908955 (PMC9486072; doi:10.3389/fpubh.2022.908955)
Supplement: Supplementary file 1 [file Data_Sheet_1.docx]

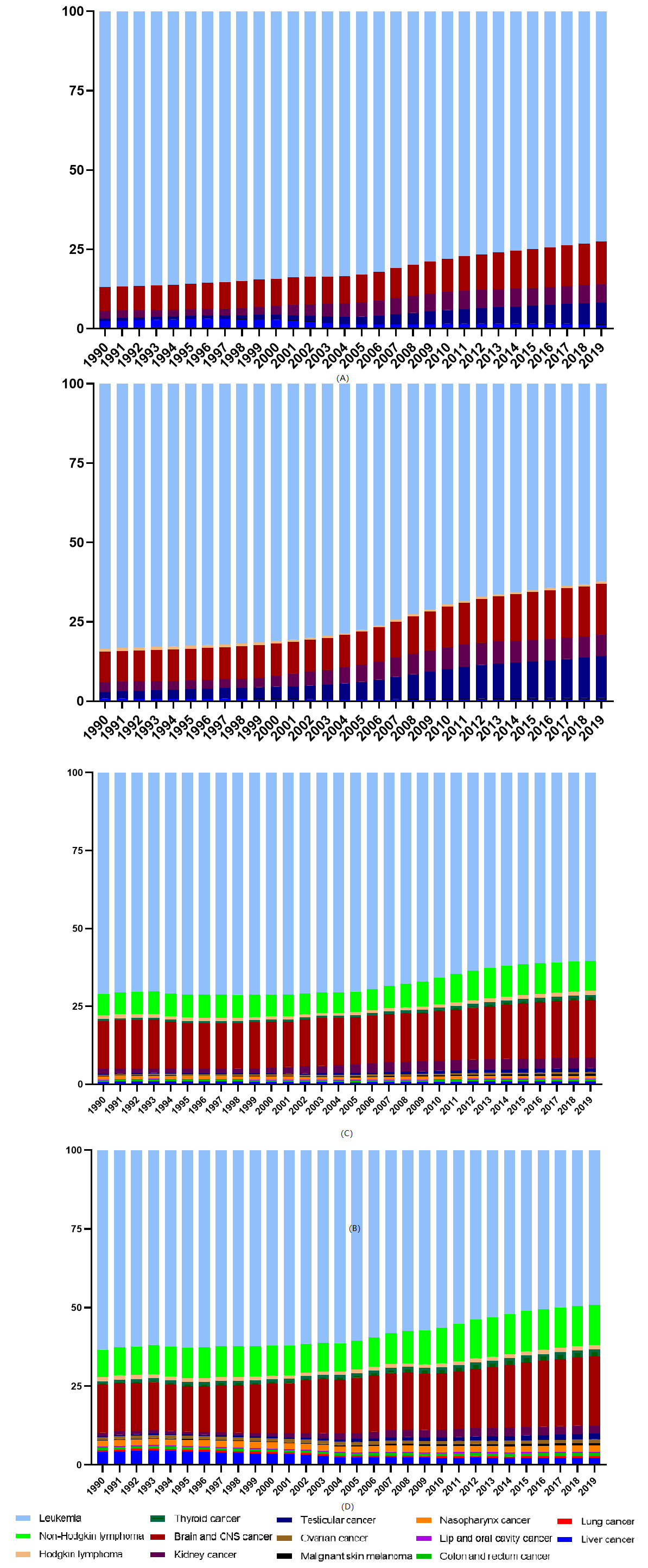


Figure 1S. The changes in the proportion of new cases of childhood cancers in four age groups in China from 1990 to 2019

1. The changes in the proportion of new cases of childhood cancers in <1 year age group in China from 1990 to 2019
2. The changes in the proportion of new cases of childhood cancers in 1 to 4 age group in China from 1990 to 2019
3. The changes in the proportion of new cases of childhood cancers in the 5 to 9 age group in China from 1990 to 2019
4. The changes in the proportion of new cases of childhood cancers in the 10 to 14 age group in China from 1990 to 2019


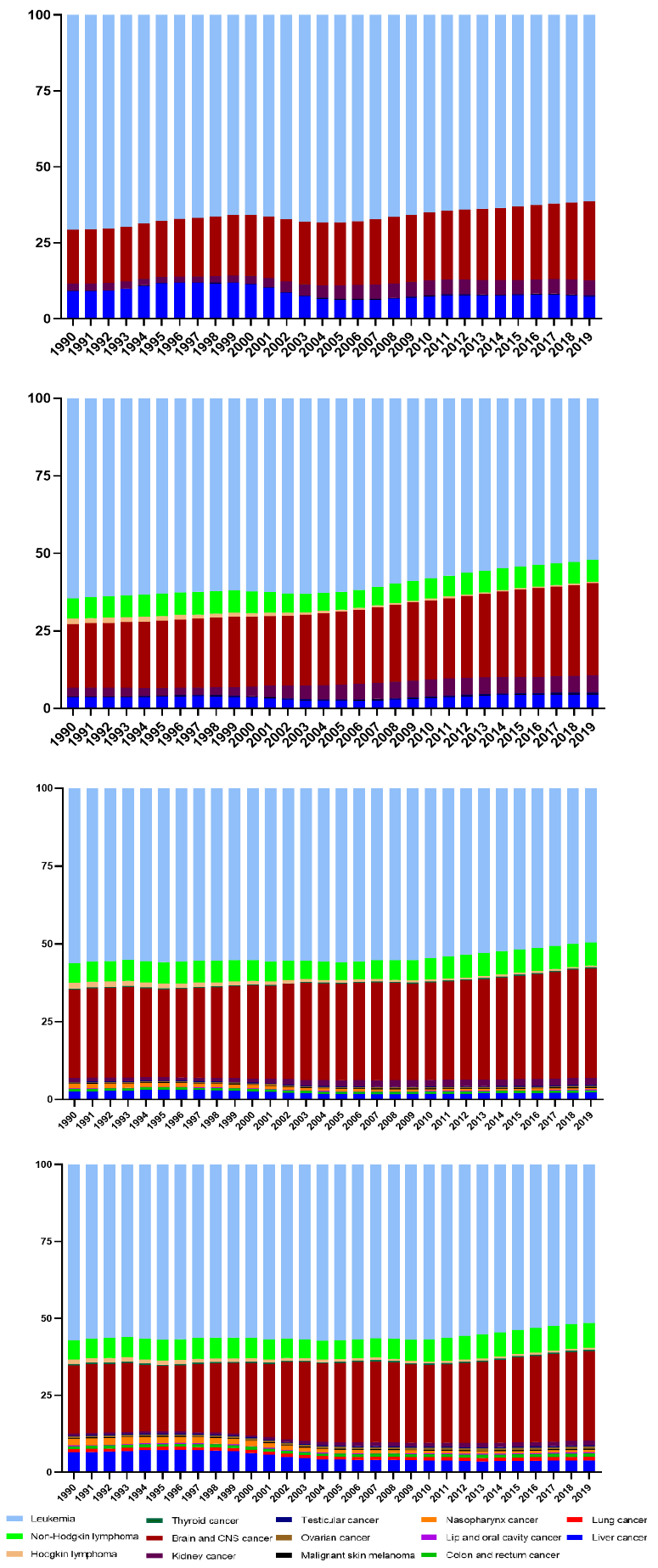


Figure 2S. The changes in the proportion of cancer deaths of childhood cancers in four age groups in China from 1990 to 2019

1. The changes in the proportion of cancer deaths of childhood cancers in <1 year age group in China from 1990 to 2019
2. The changes in the proportion of cancer deaths of childhood cancers in 1 to 4 age group in China from 1990 to 2019
3. The changes in the proportion of cancer deaths of childhood cancers in 5 to 9 age group in China from 1990 to 2019
4. The changes in the proportion of cancer deaths of childhood cancers in the 10 to 14 age group in China from 1990 to 2019


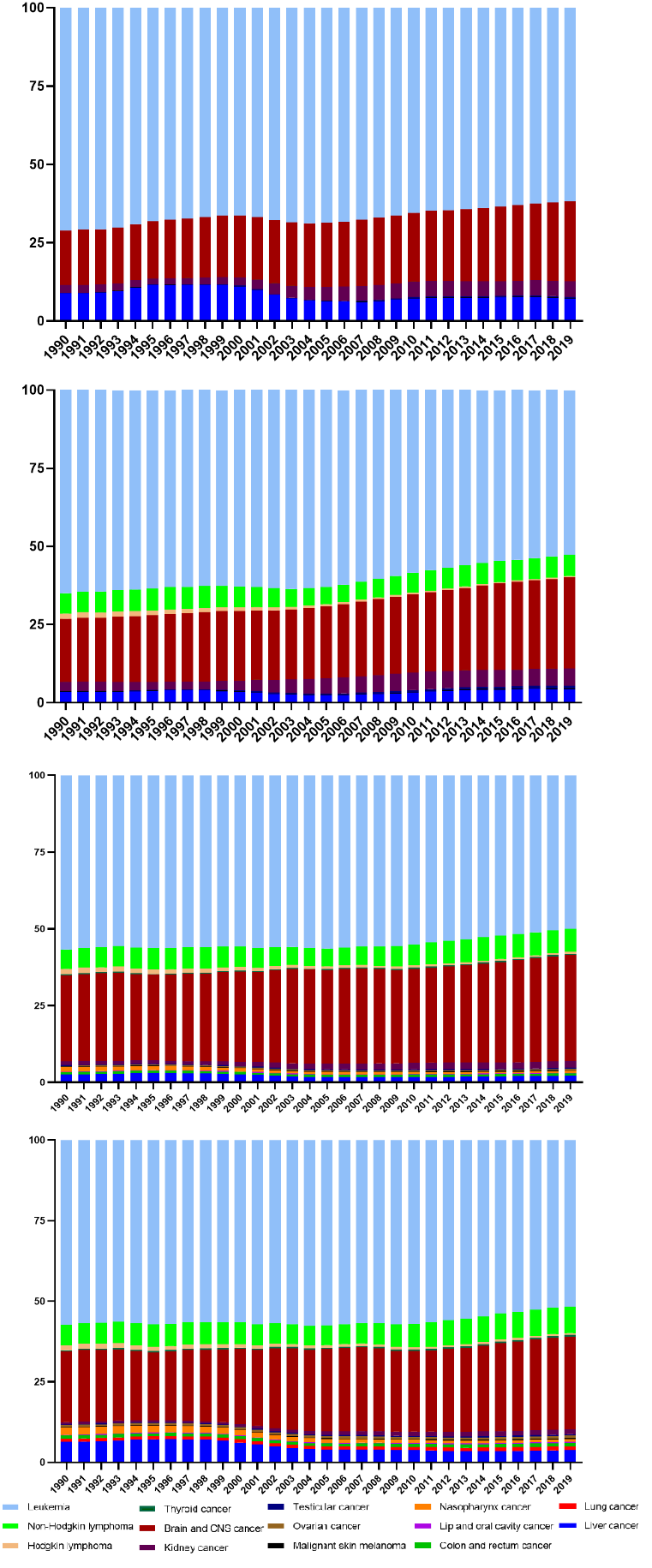


Figure 3S. The changes in the proportion of DALYs of childhood cancers in four age groups in China from 1990 to 2019

1. The changes in the proportion of DALYs of childhood cancers in <1 year age group in China from 1990 to 2019
2. The changes in the proportion of DALYs of childhood cancers in 1 to 4 age group in China from 1990 to 2019
3. The changes in the proportion of DALYs of childhood cancers in the 5 to 9 age group in China from 1990 to 2019
4. The changes in the proportion of DALYs of childhood cancers in the 10 to 14 age group in China from 1990 to 2019


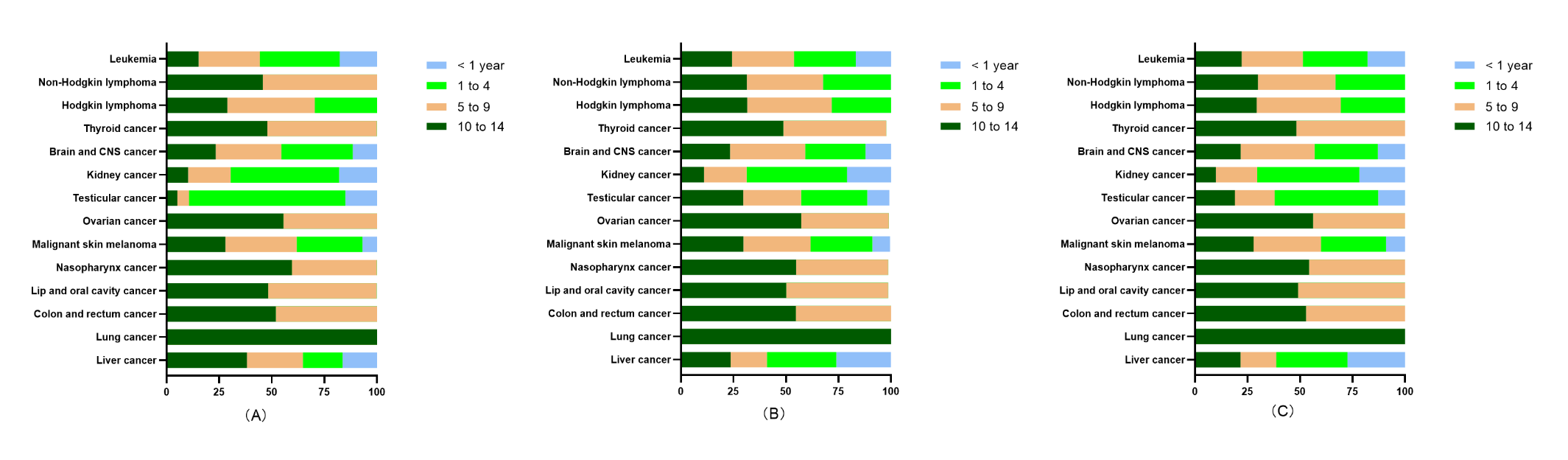


Figure 4S. The distribution of new cases, cancer deaths, and DALYs of childhood cancers in four age groups in China in 2019

1. The distribution of new cases of childhood cancers in four age groups in China in 2019
2. The distribution of cancer deaths of childhood cancers in four age groups in China in 2019
3. The distribution of DALYs of childhood cancers in four age groups in China in 2019

Table 1S. The number of new cases, cancer deaths, and DALYs of childhood cancers from 1990 to 2019 in China in China and Globally.

|  | **China** | |  | **Globally** | |  |
| --- | --- | --- | --- | --- | --- | --- |
| **Cases(No.)** | **1990** | **2019** | **1990-2019 increase (%)** | **1990** | **2019** | **1990-2019 increase (%)** |
| Leukemia | 82695 | 27727 | -66.47 | 203439 | 106082 | -47.86 |
| Brain and CNS cancer | 11509 | 7966 | -30.78 | 41071 | 39933 | -2.77 |
| Non-Hodgkin lymphoma | 2715 | 2372 | -12.63 | 13450 | 14793 | 9.99 |
| Kidney cancer | 2365 | 2223 | -6.00 | 11171 | 12291 | 10.03 |
| Liver cancer | 1732 | 504 | -70.90 | 4735 | 3719 | -21.46 |
| Testicular cancer | 1193 | 3010 | 152.31 | 11707 | 19053 | 62.76 |
| Hodgkin lymphoma | 834 | 415 | -50.24 | 4575 | 4613 | 0.83 |
| Nasopharynx cancer | 468 | 294 | -37.18 | 1232 | 1120 | -9.10 |
| Thyroid cancer | 298 | 404 | 35.57 | 1563 | 2356 | 50.76 |
| Ovarian cancer | 237 | 188 | -20.68 | 1460 | 2512 | 72.09 |
| Colon and rectum cancer | 223 | 168 | -24.66 | 1030 | 1046 | 1.53 |
| Malignant skin melanoma | 99 | 218 | 120.20 | 1287 | 1829 | 42.19 |
| Lip and oral cavity cancer | 90 | 71 | -21.11 | 810 | 1086 | 33.96 |
| Tracheal, bronchus, and lung cancer | 84 | 40 | -52.38 | 286 | 281 | -1.59 |
| Total | 104543 | 45602 | -56.38 | 297815 | 210715 | -29.25 |
| **Deaths(No.)** |  |  |  |  |  |  |
| Leukemia | 22275 | 4806 | -78.42 | 69668 | 34371 | -50.66 |
| Brain and CNS cancer | 7779 | 2816 | -63.80 | 26219 | 19988 | -23.76 |
| Liver cancer | 1835 | 368 | -79.95 | 4939 | 3290 | -33.39 |
| Non-Hodgkin lymphoma | 1732 | 584 | -66.28 | 8102 | 5825 | -28.10 |
| Kidney cancer | 712 | 313 | -56.04 | 3205 | 2713 | -15.37 |
| Hodgkin lymphoma | 452 | 42 | -90.71 | 2370 | 1728 | -27.10 |
| Nasopharynx cancer | 263 | 32 | -87.83 | 724 | 438 | -39.50 |
| Colon and rectum cancer | 123 | 46 | -62.60 | 561 | 469 | -16.45 |
| Tracheal, bronchus, and lung cancer | 66 | 27 | -59.09 | 221 | 209 | -5.15 |
| Testicular cancer | 61 | 30 | -50.82 | 510 | 527 | 3.18 |
| Ovarian cancer | 61 | 31 | -49.18 | 368 | 538 | 46.20 |
| Thyroid cancer | 52 | 22 | -57.69 | 215 | 199 | -7.53 |
| Malignant skin melanoma | 47 | 24 | -48.94 | 310 | 290 | -6.54 |
| Lip and oral cavity cancer | 29 | 15 | -48.28 | 288 | 337 | 16.74 |
| Total | 37477 | 9156 | -70.18 | 119690 | 72940 | -39.06 |
| **DALYs(No.)** |  |  |  |  |  |  |
| Leukemia | 1923732 | 415045 | -78.43 | 5945931 | 2905011 | -51.14 |
| Brain and CNS cancer | 653056 | 236113 | -63.84 | 2199198 | 1666432 | -24.23 |
| Testicular cancer | 5487 | 4043 | -26.32 | 48114 | 53259 | **10.69** |
| Non-Hodgkin lymphoma | 144261 | 48915 | -66.09 | 674140 | 482430 | -28.44 |
| Kidney cancer | 62062 | 27660 | -55.43 | 277200 | 234677 | -15.34 |
| Liver cancer | 155191 | 30890 | -80.10 | 416617 | 274538 | -34.10 |
| Hodgkin lymphoma | 37543 | 3671 | -90.22 | 192226 | 139275 | -27.55 |
| Thyroid cancer | 4271 | 1910 | -55.27 | 17765 | 16865 | -5.07 |
| Nasopharynx cancer | 20931 | 2638 | -87.40 | 57408 | 34736 | -39.49 |
| Malignant skin melanoma | 3944 | 2070 | -47.53 | 26472 | 24833 | -6.19 |
| Ovarian cancer | 4869 | 2503 | -48.60 | 29541 | 43336 | **46.70** |
| Colon and rectum cancer | 9827 | 3763 | -61.71 | 45121 | 37651 | -16.56 |
| Lip and oral cavity cancer | 2361 | 1202 | -49.08 | 23085 | 26908 | **16.56** |
| Tracheal, bronchus, and lung cancer | 5057 | 2106 | -58.35 | 16941 | 16066 | -5.17 |
| Total | 3032592 | 782530 | -74.20 | 9969759 | 5956016 | -40.26 |

Table 2S. The incidence, mortality, and DALYs rates of childhood cancers from 1990 to 2019 in China and Globally.

|  | **China** | |  | **Globally** | |  |
| --- | --- | --- | --- | --- | --- | --- |
| **The Incidence rate（per 100,000）** | **1990** | **2019** | **AAPC(95%CI)** | **1990** | **2019** | **AAPC(95%CI)** |
| Leukemia | 25.61 | 12.34 | -2.93 (-3.14 to -2.72) | 11.60 | 5.41 | -2.78 (-2.89 to -2.67) |
| Brain and CNS cancer | 3.56 | 3.54 | -0.36 (-0.71 to -0.02) | 2.34 | 2.04 | -0.53 (-0.64 to -0.43) |
| Testicular cancer | 0.37 | 1.34 | 4.92 (4.51 to 5.32) | 0.67 | 0.97 | 1.53 (1.43 to 1.62) |
| Non-Hodgkin lymphoma | 0.84 | 1.06 | 0.32 (-0.27 to 0.91) | 0.77 | 0.75 | -0.27 (-0.41 to -0.12) |
| Kidney cancer | 0.73 | 0.99 | 1.92 (1.45 to 2.40) | 0.64 | 0.63 | 0.23 (0.09 to 0.36) |
| Liver cancer | 0.54 | 0.22 | -4.18 (-4.93 to -3.42) | 0.27 | 0.19 | -1.63 (-1.90 to -1.37) |
| Hodgkin lymphoma | 0.26 | 0.18 | -1.69 (-2.24 to -1.14) | 0.26 | 0.24 | -0.42 (-0.56 to -0.28) |
| Thyroid cancer | 0.09 | 0.18 | 1.99 (1.42 to 2.57) | 0.09 | 0.12 | 0.85 (0.71 to 0.99) |
| Nasopharynx cancer | 0.14 | 0.13 | -1.12 (-1.67 to -0.57) | 0.07 | 0.06 | -1.27 (-1.54 to -1.00) |
| Malignant skin melanoma | 0.03 | 0.10 | 4.48 (4.13 to 4.83) | 0.07 | 0.09 | 0.94 (0.88 to 0.99) |
| Ovarian cancer | 0.07 | 0.08 | -0.40 (-0.92 to 0.12) | 0.08 | 0.13 | 1.32 (1.23 to 1.41) |
| Colon and rectum cancer | 0.07 | 0.07 | -0.17 (-0.63 to 0.28) | 0.06 | 0.05 | -0.61 (-0.74 to -0.47) |
| Lip and oral cavity cancer | 0.03 | 0.03 | 0.12 (-0.14 to 0.39) | 0.05 | 0.06 | 0.54 (0.48 to 0.60) |
| Tracheal, bronchus, and lung cancer | 0.03 | 0.02 | -1.76 (-2.36 to -1.16) | 0.02 | 0.01 | -0.71 (-0.92 to -0.51) |
| Overall rate | 32.38 | 20.29 | -0.34 (-0.41 to -0.27) | 16.99 | 10.75 | -0.47 (-0.54 to -0.40) |
| **The Mortality rate（per 100,000）** |  |  |  |  |  |  |
| Leukemia | 6.90 | 2.14 | -4.31 (-4.54 to -4.08) | 3.97 | 1.75 | -2.89 (-2.98 to -2.80) |
| Brain and CNS cancer | 2.41 | 1.25 | -2.72 (-3.01 to -2.43) | 1.49 | 1.02 | -1.40 (-1.49 to -1.31) |
| Testicular cancer | 0.02 | 0.01 | -1.60 (-1.79 to -1.40) | 0.03 | 0.03 | -0.08 (-0.15 to -0.01) |
| Non-Hodgkin lymphoma | 0.54 | 0.26 | -3.10 (-3.66 to -2.54) | 0.46 | 0.30 | -1.54 (-1.67 to -1.42) |
| Kidney cancer | 0.22 | 0.14 | -0.98 (-1.35 to -0.60) | 0.18 | 0.14 | -0.71 (-0.83 to -0.58) |
| Liver cancer | 0.57 | 0.16 | -5.45 (-6.34 to -4.55) | 0.28 | 0.17 | -2.14 (-2.45 to -1.83) |
| Hodgkin lymphoma | 0.14 | 0.02 | -7.78 (-8.27 to -7.28) | 0.14 | 0.09 | -1.47 (-1.63 to -1.31) |
| Thyroid cancer | 0.02 | 0.01 | -2.25 (-2.79 to -1.70) | 0.01 | 0.01 | -0.83 (-0.95 to -0.70) |
| Nasopharynx cancer | 0.08 | 0.01 | -7.40 (-8.09 to -6.71) | 0.04 | 0.02 | -2.63 (-2.89 to -2.37) |
| Malignant skin melanoma | 0.01 | 0.01 | -1.23 (-1.42 to -1.04) | 0.02 | 0.01 | -0.51 (-0.59 to -0.42) |
| Ovarian cancer | 0.02 | 0.01 | -2.08 (-2.65 to -1.51) | 0.02 | 0.03 | 0.77 (0.67 to 0.86) |
| Colon and rectum cancer | 0.04 | 0.02 | -2.73 (-3.22 to -2.23) | 0.03 | 0.02 | -1.28 (-1.41 to -1.15) |
| Lip and oral cavity cancer | 0.01 | 0.01 | -1.48 (-1.77 to -1.19) | 0.02 | 0.02 | 0.07 (-0.01 to 0.15) |
| Tracheal, bronchus, and lung cancer | 0.02 | 0.01 | -2.22 (-2.83 to -1.60) | 0.01 | 0.01 | -0.84 (-1.05 to -0.63) |
| Overall rate | 10.99 | 4.07 | -3.75 (-4.05 to -3.46) | 6.70 | 3.62 | -1.91 (-2.02 to -1.81) |
| **The DALYs rate（per 100,000）** |  |  |  |  |  |  |
| Leukemia | 595.75 | 184.65 | -4.31 (-4.54 to -4.09) | 339.00 | 148.24 | -2.92 (-3.01 to -2.83) |
| Brain and CNS cancer | 202.24 | 105.04 | -2.72 (-3.02 to -2.43) | 125.38 | 85.03 | -1.41 (-1.51 to -1.32) |
| Testicular cancer | 1.70 | 1.80 | -0.04 (-0.25 to 0.17) | 2.74 | 2.72 | 0.17 (0.10 to 0.25) |
| Non-Hodgkin lymphoma | 44.68 | 21.76 | -3.08 (-3.64 to -2.51) | 38.44 | 24.62 | -1.55 (-1.69 to -1.42) |
| Kidney cancer | 19.22 | 12.31 | -0.92 (-1.30 to -0.54) | 15.80 | 11.97 | -0.70 (-0.83 to -0.57) |
| Liver cancer | 48.06 | 13.74 | -5.46 (-6.35 to -4.55) | 23.75 | 14.01 | -2.17 (-2.48 to -1.85) |
| Hodgkin lymphoma | 11.63 | 1.63 | -7.65 (-8.16 to -7.14) | 10.96 | 7.11 | -1.49 (-1.65 to -1.32) |
| Thyroid cancer | 1.32 | 0.85 | -1.97 (-2.51 to -1.42) | 1.01 | 0.86 | -0.74 (-0.87 to -0.61) |
| Nasopharynx cancer | 6.48 | 1.17 | -7.23 (-7.90 to -6.55) | 3.27 | 1.77 | -2.63 (-2.90 to -2.37) |
| Malignant skin melanoma | 1.22 | 0.92 | -1.10 (-1.31 to -0.89) | 1.51 | 1.27 | -0.48 (-0.57 to -0.40) |
| Ovarian cancer | 1.51 | 1.11 | -2.03 (-2.59 to -1.47) | 1.68 | 2.21 | 0.78 (0.69 to 0.87) |
| Colon and rectum cancer | 3.04 | 1.67 | -2.67 (-3.15 to -2.17) | 2.57 | 1.92 | -1.29 (-1.42 to -1.15) |
| Lip and oral cavity cancer | 0.73 | 0.53 | -1.44 (-1.72 to -1.16) | 1.32 | 1.37 | 0.06 (-0.02 to 0.14) |
| Tracheal, bronchus, and lung cancer | 1.57 | 0.94 | -2.22 (-2.83 to -1.60) | 0.97 | 0.82 | -0.84 (-1.05 to -0.64) |
| Overall rate | 939.15 | 348.13 | -3.74 (-4.03 to -3.44) | 568.40 | 303.92 | -1.93 (-2.04 to -1.82) |

Table 3S. The MI values of childhood cancers from 1990 to 2019 in China and Globally.

|  | China | |  | Globally | |  |
| --- | --- | --- | --- | --- | --- | --- |
|  | 1990 | 2019 | 1990-2019 increase (%) | 1990 | 2019 | 1990-2019 increase (%) |
| Leukemia | 0.27 | 0.17 | -37.04 | 0.34 | 0.32 | -5.88 |
| Brain and CNS cancer | 0.68 | 0.35 | -48.53 | 0.64 | 0.50 | -21.88 |
| Testicular cancer | 0.05 | 0.01 | -80.00 | 0.04 | 0.03 | -25.00 |
| Non-Hodgkin lymphoma | 0.64 | 0.25 | -60.94 | 0.60 | 0.40 | -33.33 |
| Kidney cancer | 0.30 | 0.14 | -53.33 | 0.28 | 0.22 | -21.43 |
| Liver cancer | 1.06 | 0.73 | -31.13 | 1.04 | 0.89 | -14.42 |
| Hodgkin lymphoma | 0.54 | 0.11 | -79.63 | 0.54 | 0.38 | -29.63 |
| Thyroid cancer | 0.22 | 0.06 | -72.73 | 0.11 | 0.08 | -27.27 |
| Nasopharynx cancer | 0.57 | 0.08 | -85.96 | 0.57 | 0.33 | -42.11 |
| Malignant skin melanoma | 0.33 | 0.10 | -69.70 | 0.29 | 0.11 | -62.07 |
| Ovarian cancer | 0.29 | 0.13 | -55.17 | 0.25 | 0.23 | -8.00 |
| Colon and rectum cancer | 0.57 | 0.29 | -49.12 | 0.50 | 0.40 | -20.00 |
| Lip and oral cavity cancer | 0.33 | 0.33 | 0.00 | 0.40 | 0.33 | -17.50 |
| Tracheal, bronchus, and lung cancer | 0.67 | 0.50 | -25.37 | 0.50 | 1.00 | 100.00 |
| Total | 0.34 | 0.20 | -41.18 | 0.39 | 0.34 | -12.82 |

Table 4S. The new cases, cancer deaths, and DALYs of childhood cancers in different age groups (<1 year, 1 to 4, 5 to 9, and 10 to 14) in China in 2019

|  | New cases | | | |  | Cancer deaths | | | |  | DALYs | | | |
| --- | --- | --- | --- | --- | --- | --- | --- | --- | --- | --- | --- | --- | --- | --- |
|  | < 1 year | 1 to 4 | 5 to 9 | 10 to 14 |  | < 1 year | 1 to 4 | 5 to 9 | 10 to 14 |  | < 1 year | 1 to 4 | 5 to 9 | 10 to 14 |
| Liver cancer | 83 | 95 | 134 | 192 |  | 96 | 122 | 64 | 87 |  | 8478 | 10485 | 5237 | 6689 |
| Lung cancer | 0 | 0 | 0 | 40 |  | 0 | 0 | 0 | 27 |  | 0 | 0 | 0 | 2106 |
| Colon and rectum cancer | 0 | 0 | 81 | 87 |  | 0 | 0 | 21 | 25 |  | 0 | 0 | 1777 | 1986 |
| Lip and oral cavity cancer | 0 | 0 | 37 | 34 |  | 0 | 0 | 7 | 8 |  | 0 | 0 | 612 | 590 |
| Nasopharynx cancer | 0 | 0 | 119 | 175 |  | 0 | 0 | 14 | 18 |  | 0 | 0 | 1207 | 1431 |
| Malignant skin melanoma | 15 | 68 | 74 | 61 |  | 2 | 7 | 8 | 7 |  | 187 | 641 | 664 | 577 |
| Ovarian cancer | 0 | 0 | 84 | 104 |  | 0 | 0 | 13 | 18 |  | 0 | 0 | 1096 | 1407 |
| Testicular cancer | 452 | 2238 | 167 | 153 |  | 3 | 9 | 8 | 9 |  | 516 | 1993 | 769 | 765 |
| Kidney cancer | 400 | 1147 | 450 | 227 |  | 66 | 150 | 64 | 34 |  | 6006 | 13466 | 5453 | 2735 |
| Brain and CNS cancer | 912 | 2711 | 2482 | 1861 |  | 341 | 810 | 1006 | 660 |  | 30589 | 70996 | 83285 | 51244 |
| Thyroid cancer | 0 | 0 | 210 | 194 |  | 0 | 0 | 11 | 11 |  | 0 | 0 | 989 | 921 |
| Hodgkin lymphoma | 0 | 123 | 172 | 120 |  | 0 | 12 | 17 | 13 |  | 0 | 1123 | 1472 | 1076 |
| Non-Hodgkin lymphoma | 0 | 0 | 1288 | 1084 |  | 0 | 189 | 212 | 183 |  | 0 | 16226 | 18048 | 14642 |
| Leukemia | 4901 | 10540 | 8091 | 4195 |  | 803 | 1417 | 1418 | 1168 |  | 73954 | 128084 | 120942 | 92065 |
| Total | 6763 | 16923 | 13388 | 8528 |  | 1311 | 2715 | 2861 | 2268 |  | 119731 | 243013 | 241552 | 178234 |

Table 5S. The trend of MI values in all the childhood cancers in four age groups from 1990 to 2019

| Age group | Cancer type | MI value in 1990 | MI value in 2019 | 1990-2019 increase (%) |
| --- | --- | --- | --- | --- |
| <1 year | Liver cancer | 1.13 | 1.16 | 2.65 |
|  | Malignant skin melanoma | 0.51 | 0.13 | -74.51 |
|  | Testicular cancer | 0.03 | 0.01 | -66.67 |
|  | Kidney cancer | 0.31 | 0.16 | -48.39 |
|  | Brain and CNS cancer | 0.70 | 0.37 | -47.14 |
|  | Leukemia | 0.24 | 0.16 | -33.33 |
|  | Total | 0.30 | 0.19 | -36.67 |
| 1 to 4 | Liver cancer | 1.48 | 1.27 | -14.19 |
|  | Malignant skin melanoma | 0.49 | 0.10 | -79.59 |
|  | Testicular cancer | 0.03 | 0.004 | -85.92 |
|  | Kidney cancer | 0.30 | 0.13 | -56.67 |
|  | Brain and CNS cancer | 0.67 | 0.30 | -55.22 |
|  | Hodgkin lymphoma | 0.55 | 0.10 | -81.82 |
|  | Leukemia | 0.24 | 0.13 | -45.83 |
|  | Total | 0.29 | 0.15 | -48.28 |
| 5 to 9 | Liver cancer | 1.11 | 0.48 | -56.76 |
|  | Colon and rectum cancer | 0.52 | 0.26 | -50.00 |
|  | Lip and oral cavity cancer | 0.31 | 0.20 | -35.48 |
|  | Nasopharynx cancer | 0.54 | 0.12 | -77.78 |
|  | Malignant skin melanoma | 0.44 | 0.10 | -77.27 |
|  | Ovarian cancer | 0.24 | 0.15 | -37.50 |
|  | Testicular cancer | 0.30 | 0.05 | -83.33 |
|  | Kidney cancer | 0.28 | 0.14 | -50.00 |
|  | Brain and CNS cancer | 0.66 | 0.41 | -37.88 |
|  | Thyroid cancer | 0.17 | 0.05 | -70.59 |
|  | Hodgkin lymphoma | 0.52 | 0.10 | -80.77 |
|  | Non-Hodgkin lymphoma | 0.33 | 0.16 | -51.52 |
|  | Leukemia | 0.28 | 0.18 | -35.71 |
|  | Total | 0.36 | 0.21 | -41.67 |
| 10 to 14 | Liver cancer | 0.75 | 0.45 | -40.00 |
|  | Lung cancer | 0.78 | 0.69 | -11.54 |
|  | Colon and rectum cancer | 0.58 | 0.29 | -50.00 |
|  | Lip and oral cavity cancer | 0.34 | 0.22 | -35.29 |
|  | Nasopharynx cancer | 0.58 | 0.10 | -82.76 |
|  | Malignant skin melanoma | 0.49 | 0.12 | -75.51 |
|  | Ovarian cancer | 0.27 | 0.17 | -37.04 |
|  | Testicular cancer | 0.33 | 0.06 | -81.82 |
|  | Kidney cancer | 0.31 | 0.15 | -51.61 |
|  | Brain and CNS cancer | 0.69 | 0.35 | -49.28 |
|  | Thyroid cancer | 0.18 | 0.06 | -66.67 |
|  | Hodgkin lymphoma | 0.56 | 0.11 | -80.36 |
|  | Non-Hodgkin lymphoma | 0.34 | 0.17 | -50.00 |
|  | Leukemia | 0.43 | 0.28 | -34.88 |
|  | Total | 0.48 | 0.27 | -43.75 |
